# Monitoring and Evaluating Progress towards Universal Health Coverage in South Africa

**DOI:** 10.1371/journal.pmed.1001686

**Published:** 2014-09-22

**Authors:** John E. Ataguba, Candy Day, Di McIntyre

**Affiliations:** 1 Health Economics Unit, School of Public Health and Family Medicine, University of Cape Town, South Africa; 2 Health Systems Trust, Durban, South Africa

## Abstract

This paper is a country case study for the Universal Health Coverage Collection, organized by WHO. John E. Ataguba and colleagues illustrate progress towards UHC and its monitoring and evaluation in South Africa.

*Please see later in the article for the Editors' Summary*

This paper is part of the PLOS Universal Health Coverage Collection. This is the summary of the South Africa country case study. The full paper is available as Supporting Information file Text S1.

## Background

South Africa's colonial and apartheid inheritance is one of substantial social, economic, and health inequalities [1]. Since the first democratic elections in 1994, the human development index has declined considerably, largely because of the HIV epidemic, which has reduced life expectancy. The South African government has committed to a universal health system, which is seen as critical to improve population health and redress inequalities [2].

## Universal Health Coverage: The Policy Context

South Africa (SA) has a divided health system, with the minority of the population using private health services, particularly if covered by private voluntary health insurance (approximately 17% of the population), and the remainder of the population relying mainly on tax-funded health services [1],[3]. Many South Africans face health service access constraints.

A green paper published in late 2011 mapped out policies to move towards universal health coverage (UHC) over a 15-year period [2]. In the first phase, the emphasis is on investing in improving access to and the management and quality of public sector health services, particularly at the primary health care level. A range of activities has been initiated, driven by the very active leadership of the current minister of health. The second phase is intended to introduce a strategic purchasing mechanism, by establishing a semi-autonomous National Health Insurance Fund (NHIF). Although termed a National Health Insurance (NHI), it would be tax funded, through allocations from general tax revenue and possibly additional earmarked taxes. It is envisaged that the NHIF will create a universal entitlement to comprehensive health services, to be accessed through primary health care (PHC) gatekeepers and following referral routes.

## Monitoring and Evaluation for UHC

As SA is at an early stage in its UHC reforms, it does not have an explicit UHC monitoring and evaluation framework or system. While there are a number of administrative systems (such as the District Health Information System) and household surveys that can be used for UHC assessment, there are several challenges and deficiencies with the data [4]. Data from routine administrative systems are frequently inaccessible outside of government departments and of questionable data quality. There are also limitations in terms of equity analyses; most indicators of relevance to monitoring UHC progress can only be disaggregated by geographic area (province and sometimes district), with few indicators able to be disaggregated by other equity stratifiers, such as income and gender.

## Progress towards UHC in South Africa

Given that UHC reforms have only recently begun to be implemented, it is not feasible to assess progress in this regard. We were, however, able to assess South Africa's status relative to the goals of UHC, by drawing on some suggested international benchmarks. In relation to service inputs, SA is well below the WHO's Service Availability and Readiness Assessment (SARA) benchmark for inpatient beds (17 beds per 10,000 population in SA; 25 per 10,000 recommended by SARA). While SA is slightly above the SARA benchmark for core personnel (25 per 10,000 population in SA; SARA, 23 per 10,000), there are considerable variations in the distribution of health workers among geographic areas. There are also disparities across geographic areas in other inputs (e.g., per capita public spending on PHC services), outputs (such as utilisation rates), and health outcomes [4].

**Figure 1:**
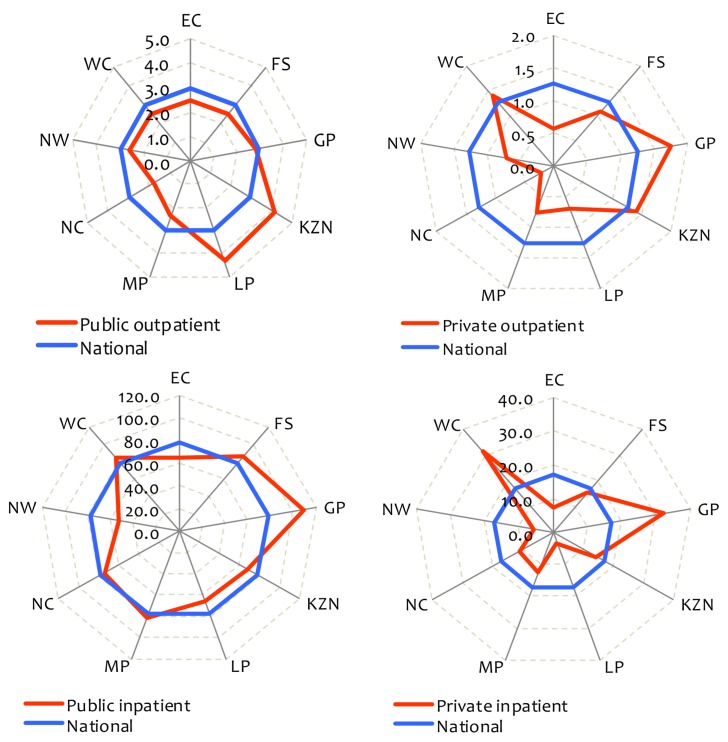
Health facility utilisation rates by province, South Africa, 2008. Outpatient and inpatient utilisation are visits per person and admissions per 1,000 population per year, respectively. The provinces are Eastern Cape (EC), Free State (FS), Gauteng (GP), KwaZulu-Natal (KZN), Limpopo (LP), Mpumalanga (MP), Northern Cape (NC), North West (NW), and Western Cape (WC). Source: Alaba and McIntyre [5].

From a UHC perspective, an indicator of particular importance is that of health service utilisation, as it provides insights into the extent to which people have access to care. The SARA benchmarks are five outpatient visits per person and 100 inpatient discharges per 1,000 population per year. While overall utilisation rates in SA appear to be in line with these benchmarks (4.2 outpatient visits and 95 inpatient admissions in either public or private facilities), Figure 1 highlights substantial differences across provinces. Utilisation rates are also lower than the SARA benchmarks for the population dependent on publicly funded services (4.1 outpatient visits and 89 inpatient admissions) yet well above these benchmarks for those with private insurance coverage (5.5 outpatient visits and 139 inpatient admissions) [5]. Disparities created by fragmented funding pools are such that the private insurance pool has per capita spending levels that are 6.2 times greater than the tax-funded pool [3].

**Figure 2:**
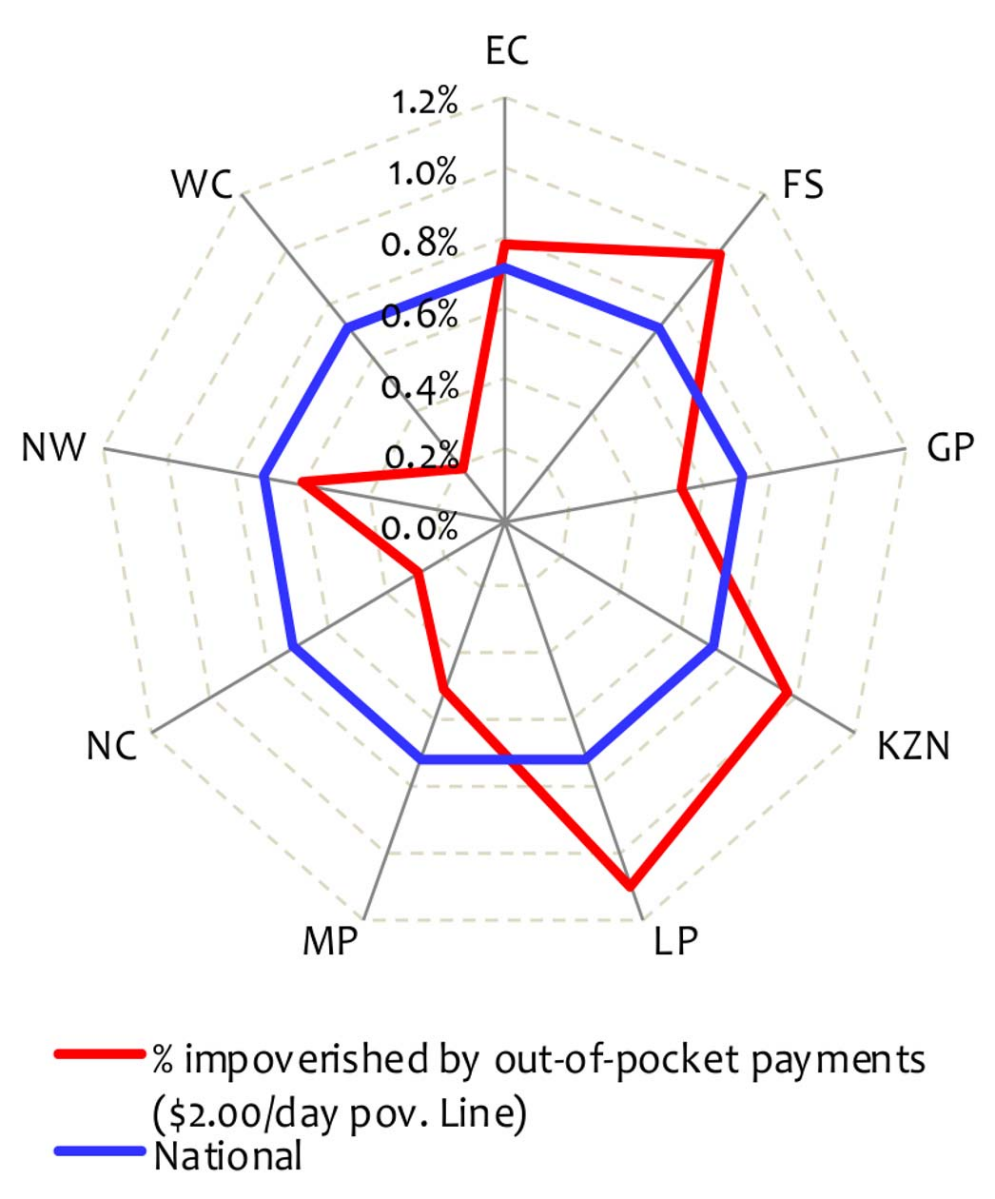
Impoverishment associated with out-of-pocket payments by province in South Africa, 2005/2006. The provinces are Eastern Cape (EC), Free State (FS), Gauteng (GP), KwaZulu-Natal (KZN), Limpopo (LP), Mpumalanga (MP), Northern Cape (NC), North West (NW), and Western Cape (WC). Source: Authors' analysis of Statistics South Africa's Income and Expenditure Survey [6].

Financial protection is also of importance from a UHC perspective. Although levels of impoverishment from out-of-pocket payments in SA are low (Figure 2), they are far greater in the poorer than richest provinces.

## Conclusions and Recommendations

To evaluate a country's status relative to UHC goals, it is critical to have UHC-related international benchmarks against which to compare country data. While some benchmarks have been suggested recently, more debate and a consensus on a widely supported set of benchmarks are needed.

It is important for SA to develop an explicit UHC monitoring and evaluation system at an early stage of reform implementation to support the refinement of reforms over time. Given its inheritance of pervasive inequalities, reducing inequalities should be emphasised while moving to UHC. Improvements in information systems and surveys are required to improve data quality, and to allow for disaggregation of indicators by a range of equity stratifiers. In addition, routine administrative data should be made more widely available.

## Supporting Information

Text S1 **The full country case study for South Africa.**
(DOCX)

## Abbreviations

SA: South Africa
SARA: Service Availability and Readiness Assessment
UHC: universal health coverage
