# Supplementary material for: Monitoring and Evaluating Progress towards Universal Health Coverage in South Africa
Source: PLoS Med. 2014 Sep 22;11(9):e1001686. doi: 10.1371/journal.pmed.1001686 (PMC4170961; doi:10.1371/journal.pmed.1001686)
Supplement: Text S1 — The full country case study for South Africa. (DOCX) [file pmed.1001686.s001.docx]

**Full Case Study: Monitoring and evaluating progress towards Universal Health Coverage in South Africa**

John E. Ataguba^1*^, Candy Day^2^ and Di McIntyre^1^

^1^ Health Economics Unit, School of Public Health and Family Medicine, University of Cape Town, South Africa

^2^ Health Systems Trust, Durban, South Africa

*Corresponding author: John E. Ataguba

Email: John.Ataguba@uct.ac.za

**This paper is the full country case study to accompany the summary paper “Monitoring and evaluating progress towards Universal Health Coverage in South Africa” that is part of the Universal Health Coverage Collection. Not commissioned; externally reviewed.**

**Abstract:** The South African health system is tiered with the minority of the population using private health services and the majority relying mainly on tax-funded health services. South Africa (SA) bears a quadruple burden of disease comprising tuberculosis, HIV and AIDS, high levels of maternal and child mortality, injuries, and non-communicable diseases. The burden of these diseases falls most heavily on the poor. In 2007 the SA government committed itself to implementing National Health Insurance (NHI) in order to move the country toward universal health coverage (UHC). This paper, as part of a series of case studies commissioned by the World Health Organization (WHO) to develop appropriate measures of UHC, provides a case study of SA’s current situation in relation to UHC using the WHO-proposed indicator framework. Drawing on different national data sources, the paper shows that disparities exist in the proposed indicators in the SA context. The paper notes that the framework may be more appropriate for monitoring progress towards UHC over time, rather than as a tool for evaluating a country’s status relative to UHC goals at a single point in time. This paper also points to the need to have UHC-related ‘benchmarks’ against which to compare country data. Further, the proposed indicators by themselves do not provide clear insights into health system reforms required to promote UHC; there is need for a more detailed system-level analysis.

**Summary Points:**

1. As South Africa is in the early stages of implementing UHC reforms, we focus on evaluating the current status of its health system relative to the goals of UHC. Such an evaluation requires a widely supported set of benchmarks against which to compare country data.

2. South Africa currently falls short of UHC goals in many respects, including its system inputs (particularly physical infrastructure), outputs (e.g. health service utilisation rates), outcomes (inadequate service coverage) and impact (poor health status). However, it does appear to have relatively good financial risk protection.

3. A key feature of South Africa is its substantial social, economic and health inequities. Monitoring reductions in inequities within the health system and health status should be prioritised.

4. An explicit UHC monitoring and evaluation system is required to support the refinement of UHC reforms over time. This requires improvements in routine information systems and surveys with respect to data quality and the range of equity stratifiers by which relevant indicators can be disaggregated.

**1. Background**

South Africa (SA) is an upper-middle income country with over 51 million people [1]. Over 60% live in urban areas. Between 2002 and 2013, the annual population growth rate was estimated at 1.3% [2] – one of the lowest in Africa [3]. With a Human Development Index (HDI) of 0.619, SA is ranked 123^rd^ out of 187 countries [4]. The HDI declined considerably from a higher value of 0.724 in 1995 (shortly after democracy) [5]. This decline is generally attributed to decreasing life expectancy associated with the increasing burden of HIV epidemic [5-7]. SA has the largest gross domestic product (GDP) in sub-Saharan Africa and accounts for about a third of the region’s total GDP [8]. However, income inequality remains high. The Gini index, a measure of income inequality, increased from 0.65 in the late 1990s to 0.72 in 2005/06 [9]. This means that the poorest 10% of the population shares about 0.1% of total incomes compared to 51% enjoyed by the richest 10% of the population. There are also correspondingly large inequalities in access to social services between population groups, provinces and socio-economic groupings [10-12]. SA bears a quadruple burden of disease, with HIV and AIDS accounting for 38% of years of life lost (YLL) in 2000, other communicable diseases and maternal and perinatal causes for 25%, non-communicable diseases (NCDs) for 21% and injuries for 16% of YLL [13]. By 2009, NCDs alone accounted for 32% of YLL [14].

Unsurprisingly given the extent of income inequality and the apartheid history, the burden of ill-health, disability and premature death falls most heavily on the poorest across most categories of disease [15]. This is even the case for some NCDs, with higher levels of self-reported hypertension among poorer groups and diabetes being relatively evenly distributed across socio-economic groups at present [15]. The poor are also affected by multimorbidity [16].

In order to address and redress these challenges, in 2007 SA committed itself to implementing National Health Insurance (NHI) to move toward universal health coverage (UHC) [17]. This requires substantial primary health care (PHC) re-engineering and other reforms. This paper therefore provides a case study of SA relative to UHC, using the indicator framework developed through a series of consultations organised by the World Health Organization (WHO). It is part of a series of country case studies, commissioned by the WHO, which are intended to contribute to the development of appropriate measures of UHC. Since there is little in the way of substantive reforms for UHC as yet in SA, with only a few initiatives such as the re-engineering of PHC and some service quality improvement initiatives currently being rolled out, it is too early to critically evaluate changes arising from these reforms. As a result, this piece does not assess progress as such but rather provides an overview of the country’s current situation. In undertaking this case study, we understand UHC to mean, ensuring that all have (i) access to, and use of, needed health care of sufficient quality to be effective; and (ii) financial protection from the costs associated with using health services [18].

**2. Universal health coverage: the policy context**

SA has a divided health system, with the minority of the population using private health services, particularly if covered by private voluntary health insurance (known as medical schemes), and the remainder of the population relying mainly on tax-funded health services.

Currently, there are about 100 different medical schemes serving about 17% of the population [19]; each scheme has an average of 4 to 5 different benefit options, each of which operates as a separate risk pool. Thus, risk pools are very fragmented in this sector. Over 60% of all out-of-pocket payments in SA are made by members of medical schemes [20]; thus membership of these schemes does not guarantee lower out-of-pocket payments [21].

There is a wide range of private for-profit providers, including: general practitioners, specialists, dentists, physiotherapists, psychologists, chiropractors and many other allied health professionals; retail pharmacies; pathology and radiology units; and private hospitals (with three large groups owning over three-quarters of private hospital beds). Private providers are particularly concentrated in the largest urban areas.

Public sector facilities consist of nationally distributed mobile and fixed clinics, and district, regional and central hospitals. A relatively comprehensive range of services (including high-cost services such as dialysis and organ transplantation) is available in public sector facilities, but there is implicit rationing through waiting lists and access problems. There are rural-urban and inter-provincial differences in the distribution of public sector facilities and other resources.

Although no fees are charged for primary care services, user fees are charged at hospitals. Pregnant women, children under 6 years and the poor are exempted from hospital fees, while other patients are charged according to three income bands. Low- and middle-income formal sector workers who are not medical scheme members have to pay the not inconsiderable fees for public sector hospitals out-of-pocket. Lack of access to public transport and lack of resources to pay for transport to health facilities make even free services prohibitive to the poorest [22].

Since the 1940s, SA has considered a series of proposals for health financing reforms but none have been fully actualised to date [23]. For example, in 1944, the National Health Services Commission (or Gluckman Commission) recommended the introduction of a national health tax to provide health services for all South Africans with no out-of-pocket payments at the point of service. Although accepted by the government, it was decided to first expand primary health care infrastructure. In 1948, a new government came to power and apartheid policies implemented, destroying any potential for pursuing a universal health system [11,24]. A series of policy recommendations put forward since the first democratic elections in 1994, primarily to introduce a mandatory health insurance scheme, were also not taken forward. The primary reason for this was opposition to introducing mandatory insurance contributions by the National Treasury (i.e. Ministry of Finance), who were concerned about placing an additional burden on middle-income groups [25,26].

In a Green Paper published in late 2011, the SA government committed itself to moving towards UHC over a 15-year period, with three five-year phases [17]. The essence of the proposed reforms is to first invest in improving access to and improved management and quality of public sector health services, particularly at the primary health care level, and then to introduce a strategic purchasing mechanism.

The focus in the first phase of reforms is to create the conditions for efficient and equitable provision of quality services within the public health system. This is necessary to reverse the damage caused by several years of declining real public funding per person dependent on public sector services from the mid-1990s, at precisely the time when demands on these services were increasing dramatically due to the HIV epidemic [11]. Although real per capita public funding has slowly increased in recent years [27], it still falls short of what is expected.

An audit of all public health facilities has been undertaken to identify where repairs, improvements and new construction are required and to ensure that each facility has a full complement of functioning equipment. Emphasis has also been placed on ensuring improved availability of medicines in all facilities. An Office of Health Standards Compliance [28] has been established to evaluate quality of care in public facilities and to put in place continuous quality improvement mechanisms. Steps are being taken to improve the management of public hospitals and health districts and gradually delegate more authority to these managers. An audit of managerial competence was undertaken, new guidelines developed on the minimum qualifications and skills required for each category of hospital, and new managers were appointed where incumbents did not meet these criteria. Several management and leadership training programs have been initiated. There are also initiatives to increase the capacity for training of nurses, doctors and other health professionals to improve staffing levels in public facilities.

A key element of the first five-year reform phase is the ‘PHC re-engineering’ program, which is seen as critical in ensuring that the public health care system will be founded on vastly improved PHC services [29,30]. Another element of strengthening PHC services is the intention to contract with private general practitioners in an effort to address human resource constraints in public sector PHC facilities.

Thus, a range of activities has been initiated to move the UHC policy agenda forward. A key factor behind the development of the reform proposals and making progress in implementing aspects of the first phase of UHC reforms has been the very active leadership provided by the current Minister of Health. This Minister was appointed in 2009, oversaw the drafting of the Green Paper, initiated and championed the ‘PHC re-engineering’ program and has vocally supported the goal of UHC.

The second five-year phase of reforms will focus on establishing the National Health Insurance Fund (NHIF) as a semi-autonomous purchaser. Although termed an NHI, it would be tax funded, through allocations from general tax revenue and possibly additional earmarked taxes (e.g. a surcharge on personal income and a payroll tax for employers). The role of the NHIF will be to pool funds and to actively purchase services from both public and private health care providers [17]. An important reason for establishing an NHIF is to be able to draw on the human and other resources located in the private provision sector to serve a greater portion of the population than currently, particularly given the severe human resource constraints in the public health sector. Another critical issue is that an NHIF would be able to overcome the constraints of the public sector financial management environment and introduce provider payment mechanisms that will incentivise the provision of efficient, high quality services. This is also important in terms of containing the current private health sector cost spiral.

It is envisaged that the NHIF will create a universal entitlement to comprehensive health services (i.e. everyone in the country will be entitled to NHIF-funded services). Services must be accessed through PHC gatekeepers and following referral routes, with an evidence-based essential drug list and standard treatment guidelines directing service delivery. It is also envisaged that private medical schemes would continue, but would play a complementary role rather than their current substitutive role.

**3. Monitoring and evaluation for UHC**

*Monitoring and evaluation framework and practices in SA*

There is no explicit monitoring and evaluation (M&E) framework for UHC in SA as yet. However, the country has a number of disease specific or program M&E frameworks including for comprehensive HIV and AIDS services, and prevention and control of NCDs. Also, a national health sector strategic plan exists under the Medium Term Strategic Framework (MTSF) for 2009-2014. The major aim of the MTSF for the health sector is to improve the health profile of all South Africans using an outcome-based approach [31]. This is envisaged through a 10-point plan for the health sector during this period. Within this, four key areas for action have been identified: increasing life expectancy; combating HIV and AIDS; decreasing the burden of disease from tuberculosis and improving health systems effectiveness [31]. This overall health sector strategic plan omits NCDs. The MTSF and other frameworks for a specific disease, program or intervention do not relate to UHC directly and as such there is no overall health system vision for monitoring UHC broadly. This is probably due to the UHC reforms being at a very early stage of implementation. There is also no specific equity dimension specified for objectives within the strategic plans (such as reducing the differential in life expectancy or infant mortality between groups). This is potentially related to the lack of an equity dimension in the Millennium Development Goals (MDGs). The MDGs have profoundly influenced the MTSF, not least of all because making progress in meeting the MDGs is a core element of the performance agreement (known as the National Service Delivery Agreement) between the Minister of Health and the South African President [29].

*Overview of indicators for monitoring progress towards UHC in SA*

In this paper, we use the broad framework proposed by the WHO [32] as the basis for assessing the SA health system in relation to UHC goals. In terms of service access, we draw on a framework that focuses on three key access dimensions: availability, acceptability and affordability [33]. The affordability dimension, closely related to financial protection, is concerned with the ‘degree of fit’ between the full costs to the individual of using a health service and the individual’s ability to pay in the context of the household budget and other demands on that budget. Availability relates to whether the appropriate health care providers and services are available in the right place and at the right time to meet the specific needs of the population. Acceptability is a frequently neglected yet critical dimension of access, which relates to the ‘degree of fit’ between provider and patient attitudes towards and expectations of each other. Frequently, the only indicators for assessing acceptability are patient satisfaction measures as opposed to issues such as mutually respectful patient-provider engagements, privacy and confidentiality, and adequate explanations of illness and discussion of treatment options, which have been shown to be key barriers to service access [34].

| **Box S1: Summary of key data sources available for assessing and monitoring UHC in SA**   - Income and Expenditure Survey (IES): Statistics SA collects detailed information on household and individual income, expenditure and other indicators of living standards, which is useful in computing financial protection indicators. From 2014, an annual Continuous Population Survey (CPS) will replace the 5-yearly IES. - Basic Accounting System (BAS): This National Treasury database provides monthly budget and expenditure information for each province according to a set of hierarchical categories such as the level of care (PHC, hospital levels, administration, emergency services), the nature of expenditure (capital, recurrent, transfers) and some broad line item categories of expenditure (human resources, goods and services). - Personnel Administration System (PERSAL): This is designed for human resources (HR) transactions and salary payments, but has limited analytical or planning capabilities for health HR. Access to the detailed records is restricted and the coding and quality of information by geographic area, level of care or occupational classification is poor - District Health Information System (DHIS): This flexible, open-source system stores aggregated health service data. In practice it collects primarily monthly data elements at facility level and selected summary indicators extracted from patient-level information systems such as the Electronic TB Register (ETR.net) and the HIV electronic register (Tier.net). Numerous authors have described varying levels of data quality problems with DHIS, based on comparison with other sources, or verification of facility records [14,54,55]. Immunisation coverage is one of the key indicators affected, and discussion of the challenges of both routine and survey based estimates of coverage have indicated that both sources have bias and quality problems [56,57]. Routine data can generally only provide facility-based indicators (e.g. maternal mortality ratio in facility) rather than population-based indicators. Though the former are important in their own right, the latter are usually required for global monitoring purposes. Despite this array of constraints the DHIS has become an increasingly useful, relatively timeous, comprehensive source of health service indicators. - Audit of health facilities: In 2011-12 an audit of all public sector facilities was conducted [47]. The assessment included the range of services provided, state of physical infrastructure, availability and functionality of equipment, human resources and systems, and the degree of compliance with national quality standards. This audit is designed to be updated on a rolling basis. This is potentially a very rich source of data on service availability and readiness for UHC. Thus far, however, very limited results have been made available from the baseline audit, and no data subsequently collected have been released. - Vital registration (cause of death) and Rapid Mortality Surveillance (RMS): Death records are the primary source for impact indicators of life expectancy and various mortality rates. The Department of Home Affairs is responsible for civil registration and maintaining a computerised national population register. The death notifications are then processed by Statistics SA and coded for causes of death. There is incomplete registration of deaths, missing data for some variables and causes of death attributed to ill-defined causes. Due to the time lag in making the cause of death data available, the RMS was developed using the national population register for more timeous calculation of some mortality indicators. The RMS thus excludes deaths not on the population register (because the deceased did not have a South African birth certificate or identity document). As the RMS data only identify cause of death as natural or unnatural, one needs to rely on the full cause-of-death data from Statistics SA to identify cause-specific death rates such as the maternal mortality ratio. In order to produce reliable indicators, various adjustments are applied to both sources of data [58,59]. - Survey data sources for assessment of service utilisation and coverage, prevalence of health conditions and risk factor exposure:   - General Household Surveys (GHS). These household surveys have been conducted annually by Statistics SA since 2002 to assess multiple facets of the living conditions and wellbeing of South African households including quality of service delivery in key service sectors of the economy including education and health [60].   - South African Consortium for Benefit Incidence Analysis (SACBIA): This collected data to address some of the limitations in GHS and the Demographic and Health Survey (DHS), among others. These data enabled the computation of reliable utilisation rates of different health services (public and private), including preventive and chronic health services. Data on the use of the different types of public facilities and private providers were also collected.   - South African DHS: Although the latest SADHS was conducted in 2003, the most reliable one was conducted in 1998. This is regarded as old and was not used to extract information for this paper.   - National Income Dynamics Study (NiDS): This survey is managed by the Southern Africa Labour and Development Research Unit (SALDRU) and is a longitudinal (panel) study to track changes over time in the livelihoods of respondents of all ages in SA. Data were collected in 2008/09, 2010/11 and 2012/13. The survey collects standard socio-demographic variables, anthropometric indicators for adults and children, as well as self-reported health status and biomarkers for certain health conditions in a representative sample of approximately 7,000 households across the country. The survey cannot be disaggregated sub-nationally, but does provide information by socio-economic status, age groups and gender. This survey was used to assess the prevalence and effective treatment coverage of hypertension. It was also used for assessing overweight and obesity in adults. It could be used to assess other NCDs and risk factors.   - South African National Health and Nutrition Examination Survey (SANHANES-1): The first wave results of this new survey were released in September 2013 [61]. The survey covers behavioural issues, nutritional status, risk behaviours and exposures and measured biomarkers for certain diseases. The survey is designed to provide national and provincial results. Although there are low response rates to aspects of the survey which may compromise results, no assessment of the survey quality is available yet. This survey is however intended to address many of the existing gaps in health indicators.   - South African national HIV prevalence, incidence, behaviour and communication surveys: Four of these household surveys have been conducted (2002, 2005, 2008 and 2012) to determine the HIV prevalence in the general population, identify risk factors that increase vulnerability of South Africans to HIV infections, to identify the contexts within which sexual behaviour occurs and the obstacles to risk reduction. The scope of the 2012 study has been expanded to include other demographic and health status indicators. |
| --- |

The main sources of data for assessing UHC in SA are summarised in Box S1. These include the Department of Health’s District Health Information System and various household surveys (including the Demographic and Health Survey, the Income and Expenditure Survey, the General Household Survey and the National Income Dynamics Study). Other administrative systems drawn on include the Basic Accounting System, the Personnel Administration System, National Health Laboratory Services and vital registration. It is beyond the scope of this paper to detail the strengths and deficiencies of each source. However, selected key challenges pertinent to measuring UHC are highlighted in Box S2.

| **Box S2: Persistent challenges with data sources for assessing UHC in SA**   - Legislative and policy environment: English et al. [62] summarise the legislation and policies pertaining to health information but note that sections of legislation have not been enacted and that overarching strategies for the harmonisation and standardisation of the health information system (HIS) have not been developed. Progress has been made with routine data, with the development of the District Health Management Information System (DHMIS) policy and the regular update of the National Indicator Data Set, although many challenges remain with implementation. - Governance and leadership challenges: There is relatively limited involvement of health system managers in data management and use for decision making and planning. Increasing emphasis on standardised frameworks for provincial and district annual health plans, and consequences from audits of reported performance is slowly improving the culture of monitoring and evaluation. - Data collection and management: Inadequate HR resources (availability and training) for data capture and management in addition to poor data collection tools, hardware and software resources affect all quality dimensions. - Integration and harmonisation: The vision of the National Health Information Repository and Database (NHIRD) is to provide up-to-date information on routine data and a unified and integrated repository for most other key data sources for health. This system holds potential but access is still restricted to a handful of government officials and success will still be limited if systematic problems with each contributing data source are not addressed. For example there is still no master facility list for the country. Geographic boundaries of provinces, districts, local municipalities and small areas have changed frequently in SA, making comparability of data over time and across sources a major challenge. Separate data sources tend to operate in isolation with poor mapping between sources. - Data quality: Quality incorporates multiple dimensions including timeliness, reliability and validity. In 2011, the Health Data Advisory and Coordination Committee (HDACC) [63] was established to improve the quality and integrity of data on health outcomes, to establish consensus on indicators – their values, measurement and mechanisms to improve data systems. HDACC has made a significant contribution, although a lot remains to be done. All data sources are beset by quality problems; although survey data sources are usually considered more rigorous and have more quality processes, they may have other biases, low response rates, missing data or other data collection problems. - Access and availability: Statistics SA, the national statistical authority, and some survey providers such as the Southern Africa Labour and Development Research Unit (SALDRU) who manage the National Income Dynamics Study (NiDS) provide full access to unit records, whereas other surveys provide only published results. Access to routine data sources tends to be limited by the time and bureaucracy of obtaining permission, and the logistics of accessing large datasets which may be provided in fragmented, inappropriate formats. - Level of representivity and disaggregation by equity stratifiers: most routine data sources tend to capture primarily public sector information, with no equivalent sources for private sector health data. Most routine systems also collect only aggregated data (i.e. monthly counts of various health service episodes). These data can be disaggregated geographically, but not according to patient characteristics. Surveys generally allow for analysis by socio-economic strata and patient characteristics for the entire population, but provide limited sub-national detail. |
| --- |

Table S1 (end of file) contains an overview of the selected indicators showing the national average (or percentage), available stratifiers, and the source of data. These representative indicators were selected based on availability and priority within the SA context and the WHO’s proposed dimensions for assessing UHC [32].

In order to show the equity dimension, a few indicators from Table S1 are disaggregated by province. The province stratifier is chosen since it is readily available for most of the selected variables and hence, allows for a more comprehensive assessment of the extent and nature of inequalities across indicators. Disaggregation by other stratifiers, such as income and gender, is only feasible for a limited number of indicators due to deficiencies in existing datasets. Although there have been substantial increases in PHC spending per capita, the pattern between provinces remains similar between 2004 and 2012 (Figure S3).

**Figure S3:** General government expenditure on PHC services per capita (real 2012/13 prices)

Data Source: Massyn et al. [42]

Note: The provinces are Eastern Cape (EC), Free State (FS), Gauteng (GP), KwaZulu-Natal (KZN), Limpopo (LP), Mpumalanga (MP), Northern Cape (NC), North West (NW), and Western Cape (WC).

Using the Income and Expenditure Survey dataset, overall health financing in SA is progressive (Figure S4); poorer households spend less as a proportion of their income on health compared to richer households. The Kakwani indices of progressivity indicate that the progressivity observed for overall financing is driven by progressive private medical scheme contributions, but these benefit only the richest in SA.

**Figure S4:** Progressivity/regressivity of health financing, South Africa, 2005/06

Data Source: Ataguba and McIntyre [43]

Note: A health financing mechanism is progressive (or regressive) when richer (poorer) households contribute a higher percentage of their income than poorer (richer) households. A negative Kakwani index represents regressivity while a positive index represents progressivity.

The distribution of medical practitioners is skewed in favour of richer provinces like the Western Cape and Gauteng possibly because they provide national (central) services while that of nurses is skewed in favour of poorer provinces (Figure S5). Generally, there is a high degree of variability in health workforce between provinces.

**Figure S5:** Health workforce density (per 100,000 population) by province, South Africa, 2012

Data Source: Personnel Administration System - reported in Day and Gray [44]

Note: The provinces are Eastern Cape (EC), Free State (FS), Gauteng (GP), KwaZulu-Natal (KZN), Limpopo (LP), Mpumalanga (MP), Northern Cape (NC), North West (NW), and Western Cape (WC).

For outputs, health facility utilisation rates for both public and private inpatient and outpatient services are shown by province in Figure S1. In general, there are inequalities in the distribution of health service utilisation with richer provinces (Western Cape and Gauteng) recording utilisation rates that are higher than the national average for these services with the exception of public outpatient services.

**Figure S1:** Health facility utilisation rate by province, South Africa, 2008

Data Source: Alaba and McIntyre [38]

Note: Outpatient and inpatient utilisation are visits per person and admissions per 1,000 population per year respectively. The provinces are Eastern Cape (EC), Free State (FS), Gauteng (GP), KwaZulu-Natal (KZN), Limpopo (LP), Mpumalanga (MP), Northern Cape (NC), North West (NW), and Western Cape (WC).

There are also variations in the distribution of other selected outcome indicators as shown in Figure S6. Stillbirth rate in facilities across the provinces show variations related to the wealth of each province as Gauteng and Western Cape record the lowest rates (Figure S7).

:

**Figure S6:** Selected health outcome indicators by province, South Africa, 2011/12 and 2012/13

Data Source: Massyn et al. [42]

Note: The provinces are Eastern Cape (EC), Free State (FS), Gauteng (GP), KwaZulu-Natal (KZN), Limpopo (LP), Mpumalanga (MP), Northern Cape (NC), North West (NW), and Western Cape (WC).


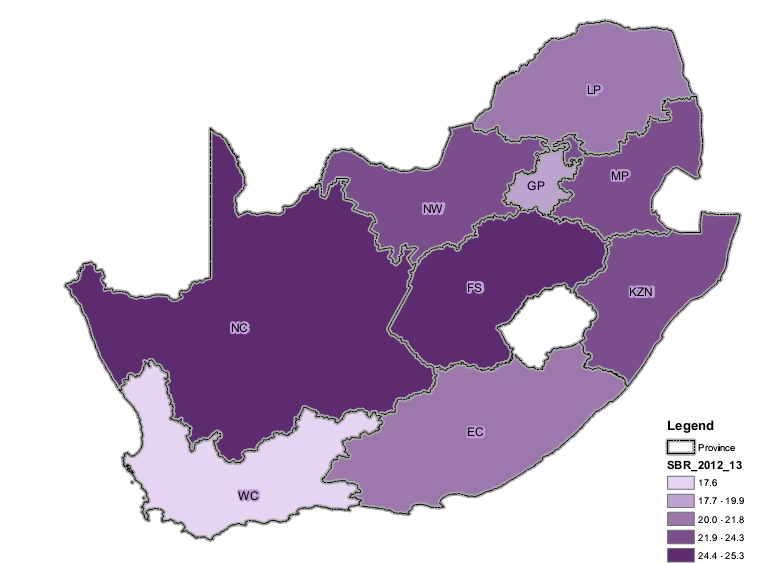


**Figure S7:** Stillbirth rate (SBR) per 1,000 births at facilities by province, South Africa, 2012/13

Data Source: Adapted from Massyn et al. [42]

Note: The provinces are Eastern Cape (EC), Free State (FS), Gauteng (GP), KwaZulu-Natal (KZN), Limpopo (LP), Mpumalanga (MP), Northern Cape (NC), North West (NW), and Western Cape (WC).

Impoverishment and financial catastrophe, although small in SA, are less prominent among the richer provinces (see Figure S2).

**Figure S2:** Impoverishment and financial catastrophe associated with out-of-pocket payments by province, South Africa, 2005/06

Data Source: Authors’ analysis based on Statistics South Africa [51]

Note: The provinces are Eastern Cape (EC), Free State (FS), Gauteng (GP), KwaZulu-Natal (KZN), Limpopo (LP), Mpumalanga (MP), Northern Cape (NC), North West (NW), and Western Cape (WC).

**4. Progress towards UHC in South Africa**

Although the national strategic plans contain some of the underlying principles of UHC, there is as yet no unified M&E framework or reporting specifically focussed on assessing UHC. UHC-related reforms have only recently begun to be implemented in SA. As a result, it is not feasible to assess progress towards UHC in this paper. It is challenging to interpret the indicators proposed by the WHO, in terms of a country’s status relative to the goal of UHC, at a single point in time unless there are ‘benchmarks’ to which they can be compared. There are currently no widely agreed UHC ‘benchmarks’, although some have recently been suggested by different groups [35-37] on which we draw here.

In relation to inputs, the WHO’s Service Availability and Readiness (SARA) initiative has a benchmark of 25 inpatient beds per 10,000 population (and SARA noted that upper-middle income countries have an average of 39), and a benchmark of 23 doctors and nurses per 10,000 population [36]. SA is well below the SARA benchmark for inpatient beds (at 17 per 10,000 population) and slightly above the benchmark for core personnel (at 25 per 10,000), although this is likely to be far lower than the average in upper-middle income countries. In relation to government health care expenditure, SA is again below the benchmark of 5% of GDP proposed by the Chatham House Working Group on Financing [37] and the goal of moving towards 5% of GDP for low- and middle-income countries proposed by the Sustainable Development Solutions Network’s (SDSN) Health Thematic Group [35].

From an outputs perspective, the SARA benchmarks are 5 outpatient visits per person and 100 inpatient discharges per 1,000 population per year [36] while the SDSN health group had more conservative benchmarks of 4 outpatient visits per person and 70 inpatient admissions per 1,000 people per year [35]. While overall utilisation rates in SA appear to be in line with these benchmarks (4.2 outpatient visits and 95 inpatient admissions in either public or private facilities), Figure S1 highlights substantial differences across provinces. Utilisation rates are also lower than the SARA benchmarks for the population dependent on publicly funded services (4.1 outpatient visits and 89 inpatient admissions) yet well above these benchmarks for those with private insurance coverage (5.5 outpatient visits and 139 inpatient admissions) [38].

In relation to outcomes, SA does not have full coverage of specific interventions. However, it is of interest that there is far higher coverage (and lower inter-provincial disparities in coverage) for interventions that have been prioritised, for example in response to the emphasis on maternal and child health and HIV/AIDS and tuberculosis in the Millennium Development Goals, than other interventions that have not received comparable prioritisation, particularly NCDs and injuries. There is an almost complete absense of indicators for NCDs, emergency services and injuries in SA. Impoverishment and catastrophic payments are not as large as in many other low- and middle-income countries, but as demonstrated in Figure S2 are greater in some provinces.

The failings of the SA health system are reflected in the impact indicators. Infant and child mortality rates in SA are higher than the SDSN benchmark of 20 per 1,000 live births while maternal mortality is more than eight times higher than the SDSN benchmark of 40 per 100,000 live births [35].

It is clear from Table S1 and the above discussion that SA has extremely poor health outcomes relative to its level of economic development and given its relatively high levels of expenditure on health (8.7% of GDP). While the HIV/AIDS epidemic has contributed to these outcomes, a similar observation was made before the HIV/AIDS epidemic began in earnest [39], suggesting more systemic problems that have not been addressed in the past two decades.

A positive aspect of the SA health system is that there appears to be relatively good financial protection in that impoverishment and catastrophic expenditure from out-of-pocket payments are low. This is in no small measure due to the removal of user fees at public sector primary health care facilities and at all public sector facilities for particularly vulnerable groups (including pregnant women, children under 6 years and the poor). While the removal of user fees improved financial access to health services, it did not remove financial barriers entirely (as the poorest often cannot afford transport costs to facilities) and the nature of the fee removal policy implementation process created other access barriers (e.g. lack of availability of medicines, health worker dissatisfaction due to increasing workloads which adversely affected patient-provider relationships)[40].

The indicator set proposed by the WHO allows some assessment of a country’s status relative to UHC goals, particularly when country results are compared to some form of UHC ‘benchmark’ levels. In the South African case, this analysis suggests that SA requires more inputs, particularly facilities and potentially more government funding, and that utilisation levels could also be improved for parts of the population. However, the indicators in general do not provide many insights into what aspects of the health system require interventions to promote UHC. A particular weakness relates to understanding barriers to accessing health services, especially in relation to the acceptability dimension [34]. The indicators that provide some insights in terms of UHC-related interventions include those with equity analyses; it is evident that inequalities across geographic areas in inputs, outputs and outcomes must be addressed. However, some data sources limit the extent of equity analyses as they lack a full range of stratifiers. In particular, while routine data can often only be stratified by geographic area, surveys usually have many other stratifiers but cannot be disaggregated by district level.

Another indicator that can potentially be of value in identifying needed health system reforms is that of pooled financial resources, given the importance of pre-payment funding for UHC. However, this indicator should be further refined to distinguish between mandatory and voluntary pre-payment mechanisms. For example, while pre-payment funds comprise about 90% of total health care expenditure in SA, almost half of these funds take the form of private voluntary insurance which only benefit 17% of the population. Another indicator included in this paper which compares per capita health care expenditure for different pools where funding pools are fragmented, highlights the large disparity in resourcing, with the private insurance pool having per capita spending levels that are 6.2 times greater than the tax-funded pool. These indicators suggest that increasing mandatory pre-payment, which the 2010 World Health Report [18] identifies as critical to achieving UHC, would be a key intervention in the SA context. The SDSN [35] in fact proposes a benchmark of voluntary funding (whether in the form of insurance or out-of-pocket payments) that should be a maximum of 30% of total expenditure; it is presently over 50% in SA.

**5. Conclusions and recommendations**

Our experience of applying the WHO indicator set to the South African context suggests that this framework may be more appropriate for monitoring progress towards UHC over time (e.g. to assess whether coverage of specific services have improved, both on average and in terms of reduced inequalities), rather than as a tool for evaluating a country’s status relative to UHC goals at a single point in time. In order to be of any value from an evaluation perspective, it is critical to have UHC-related international ‘benchmarks’ against which to compare country data. While some benchmarks have been suggested recently, it is important for there to be more debate and for a widely supported set of ‘benchmarks’ to be agreed. In addition, it needs to be recognised that these indicators by themselves do not provide clear insights into health system reforms required to promote UHC. Detailed system level analyses need to be undertaken to identify UHC reform options.

Although these indicators do not tell us the exact position of SA in relation to UHC, as SA begins to implement a wider array of UHC reforms there will be a need to monitor changes in these and other indicators over time. It is important to develop an explicit UHC monitoring and evaluation framework and system at an early stage of reform implementation to support the refinement of reforms over time to ensure that UHC goals are met. Given South Africa’s colonial and apartheid inheritance of substantial social, economic and health inequalities, it is critical that equity be a core element of this monitoring and evaluation system. In order to be able to undertake more extensive evaluation of current health system deficiencies relative to UHC goals and to monitor progress towards UHC in future, there is a need to improve, among other things, health information systems and survey data to ensure the availability of data of acceptable quality, on a wider range of indicators and that can be disaggregated by a range of relevant equity stratifiers. Also, government and other data providers should be encouraged to place datasets in the public domain and to improve linkages between different data sources to promote better monitoring of UHC.

**References**

1. Statistics South Africa (2012) Statistical release: Census 2011. Pretoria: Statistics South Africa.

2. Statistics South Africa (2013) Mid-year population estimates 2013. Pretoria: Statistics South Africa.

3. World Bank (2011) Africa Development Indicators (FACTOIDS). Washington D.C.: World Bank.

4. UNDP (2011) Human Development Report - sustainability and equity: a better future for all. New York: Palgrave Macmillan.

5. du Toit J (2002) The structure of the South African economy. Johannesburg: The SA Financial Sector Forum.

6. Cleary S, Silal S, Birch S, Carrara H, Pillay-van Wyk V, et al. (2011) Equity in the use of antiretroviral treatment in the public health care system in urban South Africa. Health Policy 99: 261-266.

7. Kahn K (2011) Population health in South Africa: dynamics over the past two decades. Journal of Public Health Policy 32: S30-S36.

8. World Bank (2011) World Development Indicators database. Washington D.C.: World Bank.

9. Statistics South Africa (2008) Income and expenditure of households 2005/2006: analysis of results. Pretoria: Statistics South Africa.

10. Mooney G, Gilson L (2009) The economic situation in South Africa and health inequities: a comment. The Lancet 374: 858-859.

11. Coovadia H, Jewkes R, Barron P, Sanders D, McIntyre D (2009) The health and health system of South Africa: historical roots of current public health challenges. The Lancet 374: 817-834.

12. Development Indicators (2012) Development indicators, 2012. Pretoria: The presidency, Republic of South Africa.

13. Bradshaw D, Groenewald P, Laubscher R, Nannan N, Nojilana B, et al. (2003) Initial Burden of Disease Estimates for South Africa, 2000. Cape Town: South African Medical Research Council.

14. Massyn N, Day C, Barron P, Haynes R, English R, et al., editors (2013) District Health Barometer 2011/12. Durban: Health Systems Trust.

15. Ataguba JE, Akazili J, McIntyre D (2011) Socioeconomic-related health inequality in South Africa: evidence from General Household Surveys. International Journal for Equity in Health 10: 48.

16. Ataguba JE (2013) Inequalities in multimorbidity in South Africa. International Journal for Equity in Health 12: 1-9.

17. Department of Health (2011) National Health Act (61/2003): Policy on National Health Insurance. Government Gazette 34523. Pretoria: National Department of Health, Republic of South Africa.

18. World Health Organization (2010) The World Health Report 2010: Health systems financing: the path to universal coverage. Geneva: World Health Organization.

19. Council for Medical Schemes (2013) Council for Medical Schemes annual report 2012-2013. Pretoria: Council for Medical Schemes.

20. McIntyre D, Doherty J, Ataguba J (2012) Health care financing and expenditure. In: Van Rensburg H, editor. Health and health care in South Africa. Pretoria: Van Schaik Publishers.

21. Ataguba JE, Goudge J (2012) The impact of health insurance on health-care utilisation and out-of-pocket payments in South Africa. The Geneva Papers on Risk and Insurance-Issues and Practice 37: 633-654.

22. Cleary S, Birch S, Chimbindi N, Silal S, McIntyre D (2013) Investigating the affordability of key health services in South Africa. Social Science & Medicine 80: 37-46.

23. Govender V, Chersich MF, Harris B, Alaba O, Ataguba JE, et al. (2013) Moving towards universal coverage in South Africa? Lessons from a voluntary government insurance scheme. Global Health Action 6: 19253.

24. McIntyre D (2010) National health insurance: providing a vocabulary for public engagement. In: Padarath A, Fonn S, editors. South African Health Review. Pretoria: Health Systems Trust.

25. McIntyre D, Doherty J, Gilson L (2003) A tale of two visions: the changing fortunes of Social Health Insurance in South Africa. Health Policy and Planning 18: 47-58.

26. McIntyre D, van den Heever A (2007) Social or national health insurance. In: Harrison S, Bhana R, Ntuli A, editors. South African Health Review 2007. Durban: Health Systems Trust. pp. 71-87.

27. Blecher M, Kollipara A, de Jager P, Zulu N (2011) Health Financing. In: Padarath A, English R, editors. South African Health Review 2011. Durban: Health Systems Trust.

28. Department of Health (2011) National Core Standards for Health Establishments in South Africa. Tshwane: National Department of Health, Republic of South Africa.

29. The Presidency (2010) Delivery Agreement for outcome 2: a long and healthy life for all South Africans. Pretoria: The presidency, Republic of South Africa.

30. Naledi T, Barron P, Schneider H (2011) Primary Health Care in SA since 1994 and implications of the new vision for PHC re-engineering. In: Padarath A, English R, editors. South African Health Review 2011. Durban: Health Systems Trust.

31. Department of Health (2010) National Department of Health Strategic Plan 2010/11-2012/13. Pretoria: South African Department of Health.

32. Evans D, Saksena P, Elovainio R, Boerma T (2013) Measuring progress towards universal health coverage. Geneva: World Health Organization.

33. McIntyre D, Thiede M, Birch S (2009) Access as a policy-relevant concept in low- and middle-income countries. Health Policy, Economics and Law 4: 179-193.

34. Bedford J, Singh A, Ponferrada MB, Eldred L (2013) Access to health services: analysing non-financial barriers in Ghana, Bangladesh, Vietnam and Rwanda using qualitative methods. A review of the literature. New York: UNICEF.

35. SDSN (2014) Health in the framework of sustainable development: technical report for the Post-2015 development agenda. New York: Thematic Group on Health for All of the Sustainable Development Solutions Network.

36. World Health Organization (2012) Measuring service availability and readiness: a health facility assessment methodology for monitoring health system strengthening. Service availability indicators. Geneva: World Health Organization.

37. Mcintyre D, Meheus F (2014) Fiscal space for domestic funding of health and other social services. London: Chatham House.

38. Alaba OA, McIntyre D (2012) What do we know about health service utilisation in South Africa? Development Southern Africa 29: 704-724.

39. McIntyre D, Bloom G, Doherty J, Brijlal P (1995) Health expenditure and finance in South Africa. Durban: Health Systems Trust and the World Bank.

40. Gilson L, McIntyre D (2005) Removing user fees for primary care in Africa: the need for careful action. British Medical Journal 331: 762-765.

41. World Health Organization (2013) World Health Statistics 2013. Geneva: World Health Organization.

42. Massyn N, Day C, Dombo M, Barron P, English R, et al., editors (2013) District Health Barometer 2012/13. Durban: Health Systems Trust.

43. Ataguba JE, McIntyre D (2012) Paying for and receiving benefits from health services in South Africa: is the health system equitable? Health Policy and Planning 27: i35-i45.

44. Day C, Gray A (2013) Health and related indicators. In: Padarath A, English R, editors. South African Health Review 2012/13. Durban: Health Systems Trust.

45. Day C, Gray A (2005) Health and related indicators. In: Ijumba P, Barron P, editors. South African Health Review 2005. Durban: Health Systems Trust.

46. Health Economics Unit, University of Cape Town, Centre for Health Policy, University of the Witwatersrand, the National Department of Health, the London School of Hygiene and Tropical Medicine (2008) South African Consortium for Benefit Incidence Analysis (SACBIA) dataset. Cape Town.

47. Health Systems Trust (2013) The national health care facilities baseline audit: national summary report, 2012. Durban: Health Systems Trust.

48. Statistics South Africa (2013) General Household Survey 2012. Pretoria: Statistics South Africa.

49. Statistics South Africa (2003) General Household Survey 2002. Pretoria: Statistics South Africa.

50. Southern Africa Labour and Development Research Unit (2012) National Income Dynamics Study 2010-2011, Wave 2. In: Southern Africa Labour and Development Research Unit, editor. Version 1 ed. Cape Town: DataFirst.

51. Statistics South Africa (2008) Income and expenditure of households 2005/2006 dataset. Pretoria: Statistics South Africa.

52. Shisana O, Rehle T, Simbayi LC, Zuma K, Jooste S, et al. (2009) South African national HIV prevalence, incidence, behaviour and communication survey 2008: a turning tide among teenagers? Cape Town: HSRC Press.

53. Bradshaw D, Dorrington RE, Laubscher R (2012) Rapid mortality surveillance report 2011. Cape Town: South African Medical Research Council.

54. Mphatswe W, Mate K, Bennett B, Ngidi H, Reddy J, et al. (2012) Improving public health information: a data quality intervention in KwaZulu-Natal, South Africa. Bulletin of the World Health Organization 90: 176-182.

55. Kawonga M, Blaauw D, Fonn S (2012) Aligning vertical interventions to health systems: a case study of the HIV monitoring and evaluation system in South Africa. Health Research Policy and Systems 10: 2.

56. Day C, Barron P, Massyn N, Padarath A, English R, et al. (2012) District Health Barometer 2010/11. Durban: Health Systems Trust.

57. Day C, Gray A, Budgell E (2011) Health and Related Indicators. In: Padarath A, English R, editors. South African Health Review 2011. Durban: Health Systems Trust.

58. Bradshaw D, Dorrington R, Laubscher R (2012) Rapid mortality surveillance report 2011. Cape Town: South African Medical Research Council.

59. Joubert J, Rao C, Bradshaw D, Dorrington RE, Vos T, et al. (2012) Characteristics, availability and uses of vital registration and other mortality data sources in post-democracy South Africa. Global Health Action 5: 19263.

60. Statistics South Africa (2008) General Household Survey 2008. Pretoria: Statistics South Africa.

61. Shisana O, Labadarios D, Rehle T, Simbayi L, Zuma K, et al. (2013) South African National Health and Nutrition Examination Survey (SANHANES-1). Cape Town: HSRC Press.

62. English R, Masilela T, Barron P, Schonfeldt A (2011) Health Information Systems in South Africa. In: Padarath A, English R, editors. South African Health Review 2011. Durban: Health Systems Trust.

63. Department of health (2012) Health Data Advisory and Co-ordination Committee (HDACC) Report. Pretoria: National Department of Health.

|  | **National average or percentage**  **(Year)** | | **Available stratifiers** | | | | | | **Data Source** |
| --- | --- | --- | --- | --- | --- | --- | --- | --- | --- |
|  |  |  | **District** | **Province** | **Household wealth or income** | **Gender** | **Urban/Rural** | **Race** |  |
| **INPUT AND PROCESS INCICATORS** | | | | | | | | | |
| **Health financing** | | | | | | | | | |
| Total health expenditure (per capita in US$) | $240.0  (2000) | $631.0  (2010) | 🗴 | 🗴 | NA | NA | 🗴 | NA | World Health Statistics from World Health Organization [41] |
| Total health expenditure (as % of GDP) | 8.1%  (2000) | 8.7%  (2010) | 🗴 | 🗴 | NA | NA | 🗴 | NA | World Health Statistics from World Health Organization [41] |
| General government expenditure on health (as % of general government expenditure) | 10.9%  (2000) | 12.4%  (2010) | 🗴 | 🗴 | NA | NA | 🗴 | NA | World Health Statistics from World Health Organization [41] |
| General government expenditure on health (as % GDP) | 3.4%  (2000) | 4.1%  (2010) | 🗴 | 🗴 | NA | NA | 🗴 | NA | World Health Statistics from World Health Organization [41] |
| General government expenditure on PHC services per capita (in SA Rand (ZAR)) – uninsured population^a^ | ZAR 324.0  (2003/04) | ZAR 780.0  (2012/13) | 🗸 | 🗸 | NA | NA | 🗴 | NA | Basic Accounting System reported in Massyn et al. [42] |
| % of household income devoted to health care financing | – | 12.8%  (2005/06) | 🗴 | 🗸 | 🗸 | 🗸 | 🗸 | 🗸 | Ataguba and McIntyre [43] using the Income and Expenditure Survey |
| **Health workforce** | | | | | | | | | |
| Pharmacists per 100,000 population (public sector) | 3.1  (2000) | 9.2  (2012) | 🗴 | 🗸 | NA | NA | 🗴 | NA | Personnel Administration System reported in Day and Gray [44,45] |
| Dental practitioners per 100,000 population (public sector) | 1.7  (2000) | 2.3  (2012) | 🗴 | 🗸 | NA | NA | 🗴 | NA | Personnel Administration System reported in Day and Gray [44,45] |
| Medical practitioners per 100,000 population (public sector) | 21.9  (2000) | 29.4  (2012) | 🗴 | 🗸 | NA | NA | 🗴 | NA | Personnel Administration System reported in Day and Gray [44,45] |
| Medical specialists per 100,000 population (public sector) | 11.2  (2000) | 11.2  (2012) | 🗴 | 🗸 | NA | NA | 🗴 | NA | Personnel Administration System reported in Day and Gray [44,45] |
| Professional nurses per 100,000 population (public sector) | 120.3  (2000) | 140.8  (2012) | 🗴 | 🗸 | NA | NA | 🗴 | NA | Personnel Administration System reported in Day and Gray [44,45] |
| Enrolled nurses per 100,000 population (public sector) | 59.7  (2000) | 69.9  (2012) | 🗴 | 🗸 | NA | NA | 🗴 | NA | Personnel Administration System reported in Day and Gray [44,45] |
| **Infrastructure** | | | |  |  |  |  |  |  |
| Hospital beds per 10,000 population | 18.04  (2008) | 17.21  (2012) | 🗸 | 🗸 | NA | NA | 🗴 | NA | District Health Barometer – Massyn et al. [14] |
|  |  |  |  |  |  |  |  |  |  |
| **OUTPUT INDICATORS** | | | | | | | | | |
| **Access: Availability** | | | | | | | | | |
| Geographic distance to facility (% within 30 minutes of health facility) | – | 75.3% (2008) | 🗴 | 🗸 | 🗸 | 🗸 | 🗸 | 🗸 | Authors’ analysis of the SACBIA data – Health Economics Unit et al. [46] |
| Tracer medicines in respect of Essential Drugs List or formulary available in the pharmacy/medicine room (clinics) (% of facilities) | – | 23.0% (2011) | 🗸 | 🗸 | NA | NA | NA | NA | The national health care facilities baseline audit reported by Health Systems Trust [47] |
| Tracer medicines in respect of Essential Drugs List or formulary available in the pharmacy/medicine room (community health centres) (% of facilities) | – | 30.0% (2011) | 🗸 | 🗸 | NA | NA | NA | NA | The national health care facilities baseline audit reported by Health Systems Trust [47] |
| Tracer medicines in respect of Essential Drugs List or formulary available in the pharmacy/medicine room (hospitals) (% of facilities) | – | 34.0% (2011) | 🗸 | 🗸 | NA | NA | NA | NA | The national health care facilities baseline audit reported by Health Systems Trust [47] |
| Functional essential medical equipment is available in the trauma/accident and emergency department (hospitals) (% of facilities) | – | 23.0% (2011) | 🗸 | 🗸 | NA | NA | NA | NA | The national health care facilities baseline audit reported by Health Systems Trust [47] |
| Functional essential medical equipment is available in the trauma/accident and emergency department (all facilities) (% of facilities) | – | 27.0% (2011) | 🗸 | 🗸 | NA | NA | NA | NA | The national health care facilities baseline audit reported by Health Systems Trust [47] |
| **Access: Acceptability** | | | | | | | | | |
| Satisfied with public healthcare facilities (%) | 81.0% (2002) | 79.2% (2012) | 🗴 | 🗸 | 🗸 | 🗸 | 🗸 | 🗸 | General Household Survey reported by Statistics South Africa [48,49] |
| Satisfied with private healthcare facilities (%) | 94.8% (2002) | 97.1% (2012) | 🗴 | 🗸 | 🗸 | 🗸 | 🗸 | 🗸 | General Household Survey reported by Statistics South Africa [48,49] |
| **Service utilisation** | | | | | | | | | |
| Age-sex standardised utilisation rate (public outpatient) – (visits per person per year) | – | 2.96 (2008) | 🗴 | 🗸 | 🗸 | NA | 🗸 | 🗸 | Alaba and McIntyre [38] using the SACBIA dataset |
| Age-sex standardised utilisation rate (public CLINIC outpatient) – (visits per person per year) | – | 2.20 (2008) | 🗴 | 🗸 | 🗸 | NA | 🗸 | 🗸 | Alaba and McIntyre [38] using the SACBIA dataset |
| Age-sex standardised utilisation rate (private outpatient) – (visits per person per year) | – | 1.27 (2008) | 🗴 | 🗸 | 🗸 | NA | 🗸 | 🗸 | Alaba and McIntyre [38] using the SACBIA dataset |
| Age-sex standardised utilisation (public inpatient) – (admissions per 1,000) | – | 78.00 (2008) | 🗴 | 🗸 | 🗸 | NA | 🗸 | 🗸 | Alaba and McIntyre [38] using the SACBIA dataset |
| Age-sex standardised utilisation (private inpatient) – (admissions per 1,000) | – | 17.00 (2008) | 🗴 | 🗸 | 🗸 | NA | 🗸 | 🗸 | Alaba and McIntyre [38] using the SACBIA dataset |
| **Pooled financial resources** | | | | | | | | | |
| % of health care expenditure through funding pools/pre-payment mechanisms versus % funding through out-of-pocket payments | 85.8% Vs. 13.3%  (2000) | 89.5% Vs. 7.4%  (2010) | 🗴 | 🗴 | 🗴 | 🗴 | 🗴 | 🗴 | World Health Organization [41] – World Health Statistics |
| Comparison of per capita health care expenditure for those covered by private insurance pools compared to those covered by the tax funded pool | – | $1370 Vs. $220  (2009) | 🗴 | 🗴 | 🗴 | 🗴 | 🗴 | 🗴 | (i) Council for Medical Schemes Annual Reports and (ii) National Treasury Budget Reviews |
|  |  |  |  |  |  |  |  |  |  |
| **OUTCOME INDICATORS** | | | | | | | | | |
| **Immunisation coverage** | | | | | | | | | |
| % children fully immunised under 1 year^b^ | 68.5%  (2002/03) | 80.1%  (2011/12) | 🗸 | 🗸 | 🗴 | 🗴 | 🗴 | 🗴 | District Health Information System – reported in Massyn et al. [42] |
| Measles 1st to 2nd dose drop-out rate (%) | 12.4%  (2002/03) | 17.0%  (2012/13) | 🗸 | 🗸 | 🗴 | 🗴 | 🗴 | 🗴 | District Health Information System – reported in Massyn et al. [42] |
| **Maternal/Reproductive health** | | | | | | | | | |
| Antenatal 1st visits before 20 weeks (%) | 30.5%  (2002/03) | 44.0%  (2012/13) | 🗸 | 🗸 | 🗴 | 🗴 | 🗴 | 🗴 | District Health Information System – reported in Massyn et al. [42] |
| Antenatal client (ANC) HIV 1st test rate (%) | 4.7%  (2002/03) | 98.0%  (2011/12) | 🗸 | 🗸 | 🗴 | 🗴 | 🗴 | 🗴 | District Health Information System – reported in Massyn et al. [14] |
| ANC initiate antiretroviral therapy (ART) rate (proxy for PMTCT coverage) (%) | – | 81.6%  (2012/13) | 🗸 | 🗸 | 🗴 | 🗴 | 🗴 | 🗴 | District Health Information System – reported in Massyn et al. [42] |
| **Other outcome indicators** | | | | | | | | | |
| TB cure rate (%) | 61.6  (2006) | 74.2%  (2011) | 🗸 | 🗸 | 🗴 | 🗸 | 🗴 | 🗴 | Electronic TB register – reported in Massyn et al. [42] |
| Hypertensives controlled on treatment (25 years and older) (%)^c^ | – | 36.4%  (2010/11) | 🗴 | 🗴 | 🗸 | 🗸 | 🗸 | 🗸 | Authors’ analysis of National Income Dynamics Study - Southern Africa Labour and Development Research Unit [50] |
| Cervical smear in woman (one smear every 10 years for women 30 years or older) (%) | 10.6%  (2002/03) | 55.4%  (2012/13) | 🗸 | 🗸 | 🗴 | 🗴 | 🗴 | 🗴 | District Health Information System (DHIS) – reported in Massyn et al. [42] |
| **Impoverishing and catastrophic payments** | | | | | | | | | |
| % of the pop impoverished by OOP - $2.00/day poverty line | – | 0.72%  (2005/06) | 🗴 | 🗸 | 🗸 | 🗸^e^ | 🗸 | 🗸 | Authors’ analysis based on the Income and Expenditure Survey from Statistics South Africa [51] |
| % of households spending over 25% of non-food expenditure OOP, unweighted | – | 0.41%  (2005/06) | 🗴 | 🗸 | 🗸 | 🗸^e^ | 🗸 | 🗸 | Authors’ analysis based on the Income and Expenditure Survey from Statistics South Africa [51] |
| % of households spending over 25% of non-food expenditure OOP, weighted | – | 0.42%  (2005/06) | 🗴 | 🗸 | 🗸 | 🗸^e^ | 🗸 | 🗸 | Authors’ analysis based on the Income and Expenditure Survey from Statistics South Africa [51] |
| **Mitigation of risk factors** | | | |  |  |  |  |  |  |
| % of the population with access to piped water^d^ | 88.6% (2001) | 91.2%  (2011) | 🗸 | 🗸 | 🗸 | 🗸 | 🗸 | 🗸 | Statistics South Africa [1] – 2011 national population census |
| % of adults overweight (15+ years) | – | 21.0%  (2010/11) | 🗴 | 🗴 | 🗸 | 🗸 | 🗸 | 🗸 | Authors’ analysis of National Income Dynamics Study - Southern Africa Labour and Development Research Unit [50] |
| % of adults obese (15+ years) | – | 37.0%  (2010/11) | 🗴 | 🗴 | 🗸 | 🗸 | 🗸 | 🗸 | Authors’ analysis of National Income Dynamics Study - Southern Africa Labour and Development Research Unit [50] |
| % of the adults 15+ using condoms | – | 62.4%  (2008) | 🗴 | 🗸 | 🗸 | 🗸 | 🗸 | 🗸 | Shisana et al. [52] - South African national HIV prevalence, incidence, behaviour and communication survey |
| TB defaulter rate (%) | 13.0%  (2000) | 8.0%  (2011) | 🗸 | 🗸 | 🗴 | 🗸 | 🗴 | 🗴 | Electronic TB register reported in Development Indicators [12] |
|  |  |  |  |  |  |  |  |  |  |
| **IMPACT INDICATORS** | | | | | | | | | |
| Life expectancy at birth (in years) | 58 years  (2000) | 60 years  (2011) | 🗴 | 🗴 | 🗴 | 🗸 | 🗴 | 🗴 | Vital Registration (for 2000) and Rapid Mortality Surveillance (for 2011) reported in Bradshaw et al. [53] |
| Infant mortality rate (per 1,000 live births) | 58  (2000) | 30  (2011) | 🗴 | 🗴 | 🗴 | 🗸 | 🗴 | 🗴 | Vital Registration (for 2000) and Rapid Mortality Surveillance (for 2011) reported in Bradshaw et al. [53] |
| U5 mortality rate (per 1,000 live births) | 70  (2000) | 42  (2011) | 🗴 | 🗴 | 🗴 | 🗸 | 🗴 | 🗴 | Vital Registration (for 2000) and Rapid Mortality Surveillance (for 2011) reported in Bradshaw et al. [53] |
| Maternal Mortality Ratio (per 100,000 live births) | – | 333 (2009) | 🗴 | 🗴 | 🗴 | NA | 🗴 | 🗴 | Statistics SA reported in Bradshaw et al. [53] |
| Stillbirth rate in facility (per 1,000 births) | 28.5  (2002/03) | 21.8  (2012/13) | 🗸 | 🗸 | 🗴 | 🗴 | 🗴 | 🗴 | District Health Information System – reported in Massyn et al. [42] |

Notes:

^a^ Average exchange rate is 1 USD = 9 ZAR

^b^ Immunisation coverage reported by DHIS is 95% for 2011/12 due primarily to the underestimation of the population under 1 year in the population denominator used by the system. The value reported here has been adjusted by using population data from the Census 2011.

c Hypertensives controlled on treatment were based on standard cut-offs for measured hypertension: systolic blood pressure of 140 mmHg or above and/or diastolic blood pressure of 90 mmHg or above.

^d^ % of population with access to piped water: Socio-economic indicators such as this tend to conceal huge disparities in service provision and ideally small-area data should be assessed, although this level of detail is only available from the census every 10 years.

^e^ Sex of household head

NA = not applicable

**Table S1:** Overview of indicators for monitoring UHC in South Africa
